# Health-related quality of life and associated factors in adults living with HIV in Rwanda

**DOI:** 10.1080/17290376.2018.1520144

**Published:** 2018-09-10

**Authors:** Juvenal Biraguma, Eugene Mutimura, José M Frantz

**Affiliations:** a College of Medicine and Health Sciences, University of Rwanda, Kigali, Rwanda; b Faculty of Community and Health Sciences, University of the Western Cape, Cape Town, South Africa; c Regional Alliance for Sustainable Development (RASD), Kigali, Rwanda

**Keywords:** Non-communicable diseases, risk factors, HIV, health-related quality of life, physical health, Rwanda

## Abstract

In Rwanda, as in other sub-Saharan African (SSA) countries, life expectancy of people living with HIV (PLWH) has increased dramatically as a result of combined antiretroviral therapy (cART). People living with HIV can now live longer but with increasing rates of non-communicable diseases (NCDs). Thus, prevention of NCD comorbidities in PWLHI is crucial to maintain and gain health-related benefits and to maximise the health-related quality of life (HRQOL) in the long-term management of PLWH. This study determines the association between physical and mental health-related dimensions of quality of life (QOL) with behavioural and biological risk factors, after controlling socio-demographic and HIV-related factors in adults living with HIV in Rwanda. A cross-sectional study using the WHO STEPwise approach and Kinyarwanda version of the MOS-HIV Health Survey, risk factors for NCDs and HRQOL were analysed for 794 PLWH, both HIV+ on ART and ART-naïve. Multiple regression analysis was used to examine the relationship between CMD risk factors and physical health and mental health summary scores. A total of 794 participants were interviewed. The mean age of the sample was 37.9 (±10.8) years and the majority of the participants were women (*n* = 513; 64.6%). About 16.2% reported daily smoking, 31.4% reported harmful alcohol use and 95% reported insufficient consumption of vegetables and fruits while 26.1% reported being physically inactive. 18.4% were overweight 43.4% had abdominal obesity, i.e. waist-hip-ratio (WHR) ≥0.95 in males and 0.85 in females. High blood pressure (HBP), i.e. systolic blood pressure (SBP) of ≥140 mmHg, or diastolic blood pressure (DBP) ≥90 mmHg was 24.4%. The results reveal that mean physical health summary and mental health summary score values were 63.96 ± 11.68 and 53.43 ± 10.89, respectively. While participants indicated that tobacco users and those who had abdominal obesity reported poor mental HRQOL, physical inactivity and hypertension have a negative impact on physical HRQOL. In addition, certain socio-demographic and HIV-related variables – specifically being unmarried, lack of HIV disclosure and low CD4 count (less 350 cell counts /mm^3^) – were associated with significantly lower mental and physical dimensions of quality of life. The results of this study reveal that behavioural and biological risk factors for NCDs were significantly associated with a lower HRQOL. These research findings also suggest that the assessment of the association between behavioural and biological risk factors for NCDs and a HRQOL provides opportunities for targeted counselling and secondary prevention efforts, so that health care providers can implement strategies that have a significant impact on the HRQOL.

## Introduction

Globally, the estimated number of PLWHI was 36.7 million [30.8 million–42.9 million] and 19.5 million were accessing antiretroviral therapy (ART) in 2016 (UNAIDS, 2017). Rwanda has achieved high rates of ART coverage, accounting 164,262 (78%) of all PLWH in 2016 and 93% of retention in care after 12 months on treatment due to the successes of Rwanda's national HIV programme (Nsanzimana et al., 2017).

Even though HIV is a chronic disease, the extensive use of ART has resulted in it being managed; available evidence supports the benefits of cART in improving HRQOL outcomes and life expectancy of those infected with HIV (Murray et al., 2014; Nakagawa, May, & Phillips, 2013). However, achieving good ART adherence is central for long-term outcomes in PLWH. Thus, identifying factors influencing ART adherence may help in understanding the strategies to enhance ART adherence for the purpose of improving HRQOL over time. However, ART does not fully restore immunity health, and a number of non-infectious diseases related to ageing and lifestyle in PLWH affect respiratory, cardiac, and endocrine systems (Dawson, Rom, Dheda, & Bateman, 2013). According to Rodriguez-Penney et al. (2013), the synergistic effects of age and HIV infection on the medical co-morbidity burden indicates that the prevalence and clinical impact of co-morbidities in older HIV+ adults underscores the importance of early detection and treatment efforts that might enhance HIV disease outcomes.

Currently, health-related benefits of PLWH on ART have shifted from the survival to HRQOL outcomes (Call et al., 2000; Miners et al., 2001). Despite the fact that the life expectancy of PLWH has increased significantly (Nakagawa et al., 2013), identification of factors influencing HRQOL in this population is critical, as QOL of PLWH may be affected by HIV progression, adverse effects of ART, and the aging process. In this case, more research is needed to gain a better understanding of the influences of socio-demographic, clinical, and psychosocial factors on HRQOL of PLWH, and of interventions which may help in improving the HRQOL in this population.

‘HRQOL’ is a term referring to the impact of disease and treatment on QOL. It is a core concept that comprises mostly self-reported measures of physical and mental health dimensions, and it has become an increasingly popular subjective health evaluation method in chronic diseases. Assessing HRQOL helps to explain the disease burden and to assess the impact and quality of the health care system in follow-up consultations with patients who have chronic diseases (Kaplan & Ries, 2007). However, it is well known that the main purpose of the HIV Care Service is to improve and strengthen QOL of PLWH. Hence, measuring HRQOL in the context of HIV infection is more important due to the chronic nature of HIV, the impact of ART, and the HIV infection itself. In this case, the HRQOL may determine the level of physical and psychological well-being of PLWH. This assessment may provide valuable information for healthcare providers and policy-makers to complement information already collected from regular clinical practices for HIV care, such as monitoring of HIV viral load and CD4 cell count, which reflect the pathological abnormalities related to HIV. Thus, a HRQOL assessment may detect the problems affecting the progression of the disease and the patients’ experiences of living with HIV, and may underscore the relevance of a multidisciplinary approach to HIV. In addition, previous researchers indicated that the physical and mental HRQOL are lower among PLWH, compared to the general population (Bing et al., 2000; Mrus et al., 2006). While earlier studies found that the physical and mental HRQOL in PLWH were poorer in comparison to people suffering from other chronic diseases (Hays et al., 2000), many researchers are currently interested in identifying HRQOL determinants with the aim of maximising the HRQOL of PLWH.

A recent review of determinants of HRQOL in PLWH revealed that HRQOL is influenced by various determinants (Degroote, Vogelaers, & Vandijck, 2014). Degroote et al. (2014) believe that there is a consensus on the influence of socio-economic status, immunological status, presence of symptoms, comorbidity, social support, and adherence to ART. Despite the growing body of knowledge regarding the determinants of HRQOL in PLWH in developing countries (Bajunirwe et al., 2009; Poupard et al., 2007; Stangl, Wamai, Mermin, Awor, & Bunnell, 2007), limited published information is available in Rwanda regarding HRQOL and HIV. A study conducted a decade ago to examine the relationship between body fat redistribution (BFR) and QOL in HAART-treated HIV+ subjects with BFR in Rwanda has reported that body fat alterations negatively affect psychological and social domains of QOL (Mutimura, Stewart, & Crowther, 2007). The study also revealed that HIV+ Rwandan women with BFR were significantly more affected by abdominal adiposity (*p* < 0 .001) and facial and buttock atrophy (*p* < 0.05) in comparison to HIV+ men with BFR. Another study by Biraguma and Rhoda (2012) assessed the prevalence of peripheral neuropathy and QOL among adults living with HIV in Rwanda. The authors found that PLWH with neuropathy had lower QOL scores in the physical and psychological domains than those without neuropathy symptoms. Evidence from these studies highlights the need for specific strategies to prevent and manage comorbidities for HIV in Rwanda.

Following this, several studies have also been conducted investigating NCD risk factors in PLWH (Edward, Oladayo, Omolola, Adetiloye, & Adedayo, 2013; Kagaruki et al., 2014; Muronya, Sanga, Talama, Kumwenda, & van Oosterhout, 2011). These studies provide evidence that risk factors for NCDs were more prevalent in PLWH on ART. This is also complemented by other researchers who found that the prevalence of hypertension was significantly higher in PLWH on ART compared to those who were not on treatment (Dimala et al., 2016; Nduka, Stranges, Sarki, Kimani, & Uthman, 2016). However, despite the widespread availability of evidence of a huge burden of NCD risk factors in PLWH, there is a dearth of research on the relationship between NCDs and the risk factors associated with them and the HRQOL in PLWH. The few available studies conducted in this area have generally focused on single risk factors for NCDs and HRQOL, and have been carried out in developed countries (Korthuis et al., 2008; Turner et al., 2001). These studies highlight the fact that participants who were physically inactive and smokers reported a lower physical and mental HRQOL. The results support integration of physical activity and smoking cessation in the clinical management of PLWH. This is also confirmed by Mutimura, Crowther, Cade, Yarasheski, and Stewart (2008) who suggest that exercise training reduces central adiposity and improves metabolic indices in HAART-treated HIV+ subjects in Rwanda. Thus, further research will help in understanding the influence of various NCD risk factors on the HRQOL of PLWH. In this regard, health care providers and policy-makers will achieve a deeper understanding of these comorbidities in the comprehensive care of PLWH. Hence**,** understanding NCD risk predictors for lower HRQOL is crucial in maximising the HRQOL in long-term management of PLWH, especially in designing health education programmes and informing health policy. The present study aims to: (1) identify physical and mental health-related dimensions of QOL among PLWH in Rwanda; and (2) determine the associations between behavioural and biological risk factors for NCDs with physical and mental health-related dimensions of QOL.

## Methodology

### Study setting, design and population

A cross-sectional quantitative design was used to collect the data. The study was conducted in randomly selected public health centres from three purposively selected provinces: Kigali City, Southern and Eastern provinces of Rwanda. The study was carried out in outpatient HIV clinics that provide HIV services. The PLWH attending the outpatients’ public health centres for receiving ARV drugs, health care consultations, counselling support as well as those who need laboratory testing, together with those who need assessment of their CD4 cell counts were included. The inclusion criteria for PLWH were all adult males and females aged over 18 years and able to provide written informed consent. Both HIV+ on ART patients and HIV+ ART naïve patients were included.

### Sampling and sample size determination

A multi-stage sampling frame was employed. Purposive sampling was employed to select two provinces; the Southern and Eastern provinces (representing the rural area) and Kigali City. Then, simple random selection of one district from each of the selected provinces, and two districts from Kigali City, was engaged. A simple random sampling of health centres was done from the lists of all health centres that offer HIV care and treatment in each selected district. The study participants were selected by using a systematic random sampling technique. Patients’ registers at the selected health centres were used to create sampling frames of all eligible patients per health centre. Sample size calculation was based on the relative contribution of eligible participants identified in each selected health centre. In this case, sample size estimation was determined using the formula *n* = Z^2^P (1-P)/d^2^ (Daniel, 1999). The researchers assumed that 50% of PLWH would have at least one of the NCD risk factors. Thus, *n* = (1.96)^2^ x0.5 (1–0.5)/ (0.05)^2^. This resulted in a minimum sample size of 384. In order to increase statistical power, to consider incomplete interviews and to incorporate a design effect within health centres, the sample was multiplied by two, as there is no previous information about design effect; this increased the sample size to 768. The sample size was also increased by 5% (38 cases) to allow for possible incomplete interviews. The representative sample size of PLWH was 806.

### Procedure

Permission was sought from the relevant authorities at the University of the Western Cape and relevant Rwandan committees. Once permission had been obtained, a team of four research assistants and the researcher visited selected health centres on different days in the period November 2014 to June 2015 based on a fixed schedule which had been arranged between the researcher and the health centre administrative staff until the sample size required at each selected health centre was reached. Each day of data collection, the researcher introduced researcher assistants to the participants prior to the start, followed by explaining the purpose of the study to the participants. After that participants voluntarily adhered to or withdrew from participating. Data collection begun by obtaining informed consent. The data were collected once off for each participant. The questionnaire was administered by research assistants who obtained face-to-face information from participants by interviewing them. Physical examination was done to obtain additional variables required.

Two validated and reliable questionnaires were used to collect data for PLWH. Data related to risk factors for NCDs were assessed based on the WHO STEPwise approach. The surveillance of NCDs risk factors was used to collect data related to risk factors for NCDs (Bonita, De Courten, Dwyer, Jamrozik, & Winkelmann, 2001). The STEP instrument covers three steps: questionnaire based-assessment, simple physical measurements and biochemical measurements. The present study utilised questionnaire assessment and physical measures only. Based on the WHO's stepwise approach, Step 1 provides information related to the questionnaire-based assessment, which includes socio-demographic characteristic data such as gender, age, marital status, educational level, employment status, monthly household income, disclosure of HIV status, residence location, tobacco use, alcohol consumption, physical activities, and diet habits related to consumption of fruit and vegetables. Information related to HIV data such as duration of awareness of HIV diagnosis, CD4 cell count, ART status, duration on ART, type of ART regimen, and adherence to ART were collected from the participants’ medical/clinical records to ensure the accuracy of the information. In Step 2 the anthropometric measurements such as weight, height, waist and hip circumference, and BP were taken.

In addition, well-trained research assistants measured and recorded the BP and anthropometric data following the WHO guidelines. Physical examinations were made using standardised techniques and calibrated equipment as described under the procedure section. An automated sphygmomanometer calibrated automatically after a pause of one minute after each BP measurement whereas the weight scales and stadiometers were calibrated at the beginning and end of each examining day. Weight, height, waist and hip circumference, and blood pressure were taken twice for each participant. The average of two measurements of each variable was considered as the procedure to minimise the random error that is associated with the measurement (Pickering et al., 2005).

The Medical Outcome Study HIV (MOS-HIV) questionnaire was used to assess the HRQOL in PLWH. The MOS-HIV questionnaire was developed from the Medical Outcomes Study (MOS), and covers 35 items grouped into 11 health dimensions including general health perception (GH), physical functioning (PF), role function (RF), social function (SF), pain (P), cognitive functioning (CF), mental health (MH), energy*/*fatigue (EF), health distress (HD), quality of life (QL) and health transition (HT). MOS-HIV is brief: it takes no longer than 10 minutes to administer. The MOS-HIV also provides an overall physical and mental health summary score with higher scores indicating better overall physical and mental summary score. The scores obtained for each dimension are transformed or standardised in order to make comparisons among various dimensions which may have different response categories. A score range was from 0, the lowest possible score, to 100, the highest score. Epino et al. (2012) indicated that the MOS-HIV has good internal consistency with a Cronbach alpha coefficient of .79 in PLWH adults in Rwanda.

### Ethical clearance

Permission and ethical approval for the study were provided by: i.) the Senate Research Grants and Study Leave Committee of the University of the Western Cape (Registration no: 13/6/34); ii.) Directorate General of Science, Technology and Research in Ministry of Education (No: MINEDUC/S&/263/214); iii.) The National Health Research Committee (Ref: NHRC/2014/PROT/0133) and iv.) The Rwanda National Ethics Committee (RNEC) (No. 248/RNEC/2014). Participants were informed of the nature and intention of the study in their language of choice. The participants were also informed of their right to withdraw from the process at any stage of the project, and that there was no harmful procedure involved. Written informed consent was obtained from volunteers before their completion the questionnaires and making use their medical records. Participation was free and voluntary. Participants were assured of complete confidentiality throughout of the study and their names will be kept anonymous. No research participant was forced to answer any questions with which they felt uncomfortable. Only those who were willing to participate were considered. In addition, females and males were accommodated in separate sections of the clinics to ensure privacy.

Data were analysed using SPSS Statistics 23. Descriptive statistics for all variables were generated. Continuous variables were summarised with mean and standard deviations. Frequency percentages summarised categorical variables. Pearson's Chi-square was used to demonstrate the association between participants’ characteristics and ART use whereas the independent-samples t-test was used to compare the mean score on physical HRQOL and mental HRQOL dimensions for the two groups of participants. Multiple regression analysis with hierarchical multiple regression was used. A hierarchical multiple regression analysis was used to assess the ability of NCDs’ risk factors to predict physical and mental health-related dimensions of QOL after controlling socio-demographic and HIV-related factors. The models included all the variables that were found to be significant in bivariate analysis.

## Results

### Socio-demographic, clinical, behavioural and biological characteristics

A total number of 806 PLWH were approached in four selected health centres, and 794 participants consented to participate, yielding a response rate of 98.5%. The mean age of the respondent was 38 years (SD = 10.8) and the ages ranged from 18 years to 70 years. The majority of the participants were female (*n* = 513; 64.6%), had primary or no formal education (*n* = 636; 80.1%), were currently married (*n* = 507; 63.9%), lived in an urban area (*n* = 535; 67.4%) and fell into the age range of 18–40 years (*n* = 485; 61.1%). Additionally, the majority of the participants had known their HIV+ status for a period of 1–6 years (*n* = 603; 76.0%), were on ART (*n* = 698; 87.9%), had their CD4+ count >350 mm^3^ (*n* = 545; 68.6%), and disclosed their HIV status (*n* = 633; 79.7%). Table 1 summarises information regarding socio-demographic and HIV-related characteristics of the study participants.

**Table 1.** Distribution of socio-demographic and HIV-related characteristics of the participants.

Characteristics	Total *N* = 794	PLWH on ART use *n* = 698	PLWH not on ART *n* = 96	*p*-value
Gender				<0.001
Women	513 (64.6)	471 (67.5)	42 (43.8)	
Men	281 (35.4)	227 (32.5)	54 (56.3)	
Age group/ years				0.570
18–30	240 (30.2)	206 (29.5)	34 (35.4)	
31–40	245 (30.9)	218 (31.2)	27 (28.1)	
41–50	204 (25.7)	183 (26.2)	21 (21.9)	
>50	105 (13.2)	91 (13.0)	14 (14.6)	
Marital status				0.076
Never married	145 (18.3)	121 (17.3)	24 (25.0)	
Currently married	507 (63.9)	457 (65.5)	50 (52.1)	
Separated/Divorced	52 (6.5)	43 (6.2)	9 (9.4)	
Widowed	90 (11.3)	77 (11.0)	13 (13.5)	
Educational level				0.378
No formal education	160 (20.2)	139 (19.9)	21 (21.9)	
Primary	476 (59.9)	415 (59.5)	61 (63.5)	
≥ Secondary	158 (19.9)	144 (20.6)	14 (14.6)	
Employment status				0.009
Public service	81 (10.2)	75 (10.7)	6 (6.3)	
Self-employed	264 (33.2)	225 (32.2)	39 (40.6)	
Peasant/Farmer	252 (31.7)	233 (33.4)	19 (19.8)	
Unemployed	197 (24.8)	165 (23.6)	32 (33.3)	
Monthly household income				0.067
≤20000 RWF	179 (22.5)	158 (22.6)	21 (21.9)	
20001–40000 RWF	311 (39.2)	276 (39.5)	35 (36.5)	
40001–60000 RWF	129 (16.2)	104 (14.9)	25 (26.0)	
60001–80000 RWF	65 (8.2)	59 (8.5)	6 (6.3)	
>80000 RWF	110 (13.9)	101 (14.5)	9 (9.4)	
Residence location				<0.001
Rural	259 (32.6)	248 (35.5)	11 (11.5)	
Urban	535 (67.4)	450 (64.5)	85 (88.5)	
Time since HIV diagnosis				0.001
≤3 years	338 (42.6)	280 (40.1)	58 (60.4)	
4–6 years	265 (33.4)	243 (34.8)	22 (22.9)	
≥7 years	191 (24.1)	175 (25.1)	16 (16.7)	
CD4 cell count				0.075
≤200 cells*/*μl	84 (10.6)	77 (11.0)	7 (7.3)	
201–350 cells*/*μl	165 (20.8)	148 (21.2)	17 (17.7)	
351–500 cells*/*μl	220 (27.7)	199 (28.5)	21 (21.9)	
>500 cells*/*μl	325 (40.9)	274 (39.3)	51 (53.1)	
Disclosure of HIV+ serostatus				<0.001
Yes	633 (79.7)	612 (87.7)	21 (21.9)	
No	161 (20.3)	86 (12.3)	75 (78.1)	

Table 2 indicates the comparison of behavioural and biological risk factors for NCDs for HIV+ participants on ART and HIV+ and ART-naïve study participants. All behavioural risk factors and BMIs were comparable, while WHR and BP were not. The proportion of the participants with increased WHR was significantly higher among PLWH on ART than PLWH not on ART (46.1% vs. 24.2%, *p* < 0.001); while the proportion of the participants with hypertension was significantly lower among ART-users than the ART-naïve participants (20.3% vs. 54.2%, *p* < 0.001).

**Table 2.** Distribution of behavioural and biological risk factors for NCDs.

Characteristics	Total *N* = 794	PLWH on ART *n* = 698	PLWH not on ART *n* = 96	*p*-value
Tobacco use				0.679
Non-users	665 (83.8)	586 (84.0)	79 (82.3)	
Users	129 (16.2)	112 (16.0)	17 (17.7)	
Alcohol use				0.795
Non-users	545 (68.6)	478 (68.5)	67 (69.8)	
Users	249 (31.4)	220 (31.5)	29 (30.2)	
Physical activity level^a^				0.276
Low	203 (26.3)	176 (25.9)	27 (29.0)	
Moderate	184 (23.8)	168 (24.7)	16 (17.2)	
High	385 (49.9)	335 (49.3)	50 (53.8)	
Fruit and vegetable intake				0.717
≥5 servings	28 (3.5)	24 (3.4)	4 (4.2)	
<5 servings	766 (96.5)	674 (96.6)	92 (95.8)	
BMI category^b^				0.056
Underweight	98 (12.3)	82 (11.7)	16 (16.7)	
Normal weight	550 (69.3)	493 (70.6)	57 (59.4)	
Overweight	115 (14.5)	100 (14.3)	15 (15.6)	
Obese	31 (3.9)	23 (3.3)	8 (8.3)	
Abdominal obesity^c^				<0.001
No	437 (56.6)	365 (53.9)	72 (75.8)	
Yes	335 (43.4)	312 (46.1)	23 (24.2)	
Hypertension^d^				<0.001
No	600 (75.6)	556 (79.7)	44 (45.8)	
Yes	194 (24.4)	142 (20.3)	52 (54.2)	

^a^Low: <600MET min/week; moderate: 600–2999 MET min/week; high: ≥1500 MET min/week vigorous physical activity or ≥ 3000 MET min/week moderate/vigorous physical activity.

^b^Underweight: <18.5; normal: 18.50–24.99; overweight: 25.00–29.99; obese: ≥ 30.0.

^c^Having WHR greater than 0.95 for men and 0.85 for women.

^d^A SBP of 140 mm Hg or more, or a DBP of 90 mm Hg or more.

### Health-related quality of life

The HRQOL was measured by the MOS-HIV Health Survey. The questionnaire consisted of 11 MOS-HIV sub-scales, including general health perception, bodily pain, physical functioning, role function, social function, mental health, vitality, health distress, cognitive functioning, QOL, and health transition. Based on these sub-scales, the PHS and MHS scores were calculated. The mean PHS value was 63.96 ± 11.68, with a range of 30–92, and the mean value of MHS was 53.43 ± 10.89, ranging from 26.0–78.3. Table 3 shows the distribution of mean, SD, median and interquartile range (IQR) values of the MOS-HIV questionnaire domains.

**Table 3.** Distribution of MOS-HIV scores.

Dimensions	Mean ± SD	Minimum	Maximum
**A. PHS**	63.96 ± 11.68	30	92
**- GH**	41.35 ± 10.28	15	70
**- PF**	67.28 ± 12.25	58	92
**- RF**	76.65 ± 36.74	0	100
**- SF**	77.34 ± 23.35	0	100
**- P**	57.04 ± 26.96	0	100
**B. MHS**	53.43 ± 10.89	26	76
**- CF**	58.15 ± 16.00	20	95
**- MH**	45.54 ± 12.05	32	76
**- EF**	54.20 ± 9.94	35	75
**- HD**	60.71 ± 19.40	15	100
**- QOL**	50.40 ± 31.74	0	100
**- HT (changes in health status)**	50.56 ± 31.93	0	100

Abbreviations: *PHS* = Physical health summary score, *GH* = General Health Perception*, PF* = Physical Functioning*, RF* = Role function*, SF* = Social function*, P* = Pain*, MHS* = Mental health summary score*, CF* = Cognitive Functioning*, MH* = Mental Health, *EF* = Energy/Fatigue*, HD* = Health Distress*, QOL* = Quality of life*, HT* = Health transition.

### Factors associated with health-related quality of life among PLWH

A hierarchical multiple regression analysis was used to assess the ability of NCD risk factors to predict overall PHS and MHS, after controlling the influence of socio demographic and HIV-related factors. Included in the model were all the variables that showed statistically significant associations (*p* < 0.05) in the bivariate analysis. Preliminary analyses were conducted to guarantee against violation of the assumptions of normality, linearity, multicollinearity, and homoscedasticity. The overall model fit revealed that socio demographics, HIV-related, and risk factors for NCDs were significantly associated with PHS and MHS.

Table 4 illustrates the association between behavioural and biological risk factors for NCDs and PHS. For PHS score dimensions the marital status, level of education, and employment status were entered into the model first, and were significantly related to PHS, explaining 8% of the variance (Table 4; R square = 0.081, *F* (3, 644) = 5.977, *p* = 0.001). Introducing CD4 cell counts, disclosure of HIV+ serostatus, and HIV duration variables added 7% of variation in the PHS, and this change in R square was significant (Table 4; R square = 0.072, *F* (3, 641) = 5.309, *p* = 0.001). The total variance explained by the model as a whole, including NCD risk factors, was 23% and significant, *F* (10, 637) = 5.168, *p* < .001 for PHS. NCD risk factors explained an additional 8% of the variance in PHS after controlling for socio demographic and HIV-related factors, and this change in R square was significant, F change (4, 637) = 4.195, *p* = 0.002. This suggested that behavioural and biological risk factors for NCDs have the effect above and beyond the effects of socio-demographic and HIV-related factors. In the final model, five control measures were statistically significant for PHS. The PHS scores increased by 0.09 points for participants who were currently married (beta = .086, *p* = .039), and decreased by 0.09 points in participants who had CD4 counts less than 350 cells/µl (beta = −.085, *p* = .036), 0.09 points in participants who had abdominal obesity (beta = −.086, *p* = .026), 0.10 for participants who were physically inactive (beta = −.098, *p* = .012), and 0.11 points for participants who were hypertensive (beta = −.107, *p* = .008).

**Table 4.** Hierarchical multiple regression analysis for predicting PHS.

Variables	PHS								
	Overall F-test	Beta	t	df	*p*-value	R square	Adjusted R square	R square change	Sig. F change
Model 1	5.977			3,644	.001	.081	.069	.081	.001
Marital status		.116	2.858		.004				
Education		.085	2.153		.032				
Employment		−.068	−1.641		.101				
Model 2	5.703			6,641	.000	.153	.126	.072	.001
Marital status		.104	2.525		.012				
Education		.071	1.822		.069				
Employment		−.052	−1.256		.210				
CD4 count		−.110	−2.731		.006				
Disclosure of HIV+ serostatus		−.027	−.639		.523				
HIV duration		−.063	−1.609		.108				
Model 3	5.168			10,637	.000	.225	.183	.072	.002
Marital status		.086	2.038		.039				
Education		.070	1.788		.074				
Employment		−.033	−.793		.428				
CD4 count		−.085	−2.015		.036				
Disclosure of HIV+ serostatus		.001	.012		.990				
HIV duration		−.065	−1.642		.101				
Tobacco use		−.053	−1.106		.269				
Physical inactivity		−.098	−2.374		.012				
Abdominal obesity		−.086	−2.225		.026				
Hypertension		−.107	−2.421		.008				

F-statistic and *p*-value <0.05.

Table 5 shows the association between behavioural and biological risk factors for NCDs and MHS. For MHS dimensions the age, marital status, and employment status were entered in the model first, and were significantly related to MHS, explaining 9% of the variance (Table 5; R square = 0.090, *F* (3, 722) = 7.418, *p* < .001). Introducing CD4 cell counts and disclosure of HIV+ serostatus variables explained an added 4% of variation in MHS, and this change in R square was significant (Table 5; R square = 0.042, *F* (2, 720) = 5.377, *p* = 0.05). The addition of NCDs risk factors (alcohol use, tobacco use, and abdominal obesity) at Step 3 explained an additional of 23% in the variation of MHS after controlling the influence of socio demographic and HIV-related factors, and this change in R square was significant (Table 5; R square = 0.228, *F* (3, 717) = 20.532, *p* < .001). This suggested that the relationship between NCD risk factors (alcohol use, tobacco use, and abdominal obesity) and MHS is not mediated or explained by socio- demographic and HIV-related factors. The total variance explained by the model as a whole, including NCD risk factors, was 36%, *F* (8, 717) = 12.198, *p* < .001 for MHS. The results indicate that four control measures, including marital status (beta = .111, *p* = .003), disclosure of HIV serostatus (beta = −.084, *p* = 0.027), tobacco use (beta = −.272, *p* < .001), and abdominal obesity (beta = −.089, *p* = .011) explained significant variances in the MHS. The MHS scores increased by 0.11 points for participants who were currently married, and the MHS decreased by 0.08 points for participants who did not disclose their HIV+ serostatus, decreased 0.09 points for those who had abdominal obesity, and decreased 0.27 points for those who were tobacco users.

**Table 5.** Hierarchical multiple regression analysis for predicting MHS.

Variables	MHS								
	Overall F-test	Beta	T	Df	*p*-value	R square	Adjusted R square	R square change	Sig. F change
Model 1	7.418			3,722	.000	.090	.078	.090	.000
Age		−.065	−1.767		.078				
Marital status		.135	3.470		.001				
Employment		−.051	−1.298		.195				
Model 2	6.656			5,720	.000	.132	.114	.042	.005
Age		.054	−1.445		.149				
Marital status		.114	2.925		.004				
Employment		−.029	−.744		.457				
CD4 count		−.052	−1.381		.168				
Disclosure of HIV+ serostatus		−.107	−2.755		.006				
Model 3	12.198			8,717	.000	.360	.330	.228	.000
Age		−.017	−.482		.630				
Marital status		.111	2.955		.003				
Employment		−.005	−.127		.899				
CD4 count		−.029	−.791		.429				
Disclosure of HIV+ serostatus		−.084	−2.223		.027				
Tobacco use		−.272	−7.230		.000				
Alcohol use		.008	−.225		.822				
Abdominal obesity		−.089	−2.540		.011				

F-statistic and *p*-value <0.05.

## Discussion

The study examined the association between physical and mental health-related dimensions of QOL with behavioural and biological risk factors, after controlling for socio-demographic and HIV-related factors in adults living with HIV in Rwanda. The findings revealed that the physical HRQOL dimension had higher scores than the mental HRQOL dimension in the current study. These findings are related to other studies that used a similar questionnaire (Briongos Figuero, Bachiller Luque, Palacios Martin, González Sagrado, & Eiros Bouza, 2011; Perez et al., 2005). In addition, the findings highlight an improvement in the HRQOL, contrary to a previous study (Delate & Coons, 2001). In that study (Delate & Coons, 2001), the participants reported a low HRQOL, with the mean scores for the PHS and MHS being 42.8 ± 10.9 and 46.6 ± 6.2, respectively. This confirms that the success of cART is associated with better virology and immunological status. Thus, interventions to enhance ART adherence are urgently required to improve and maintain the HRQOL of PLWH on ART. These interventions may include education, cognitive–behavioural interventions, directly observed therapy, treatment supporters, and active adherence reminder devices (Chaiyachati et al., 2014). Hence, routine assessment of the HRQOL will help to better understand the impact of HIV itself, and ART on health and the QOL of those living with HIV may provide potential information to inform interventions that can improve the HRQOL in this population. On the other hand, lower scores in the mental HRQOL may be explained by different factors, including psychosocial factors. Available evidence highlights the high prevalence of psychological morbidity, especially depression, in PLWH (Bhatia & Munjal, 2014; Mohammed, Mengistie, Dessie, & Godana, 2015), and it presents depression as an important predictor of a poor mental HRQOL (Briongos Figuero et al., 2011). In this vein, a study that aimed to assess the prevalence, socio-demographic determinants and phenomenology of depressive disorder among PLWH was conducted in Nigeria (Aguocha et al., 2016), and authors concluded that there was a high rate of depression, especially among female PLWH in South East Nigeria, and they recommended that mental health services should be an integral part of HIV care and treatment. This is also complemented by Bernard, Dabis, de Rekeneire, and Seedat (2017) who suggested that the pooled prevalence estimates of depression ranged between 9% and 32% in PLWH on ART and in untreated or mixed (treated/untreated) ones, while reported factors of depression were low socio-economic conditions in PLWH on ART, female sex and immunosuppression in mixed/untreated PLWH. In this regard, early identification and proper treatment of psychological morbidity may enhance the mental HRQOL of PLWH. Thus, routine screening and treating depression in PLWH are highly recommended.

The results of this study reveal that behavioural and biological risk factors for NCDs were significantly associated with a lower HRQOL. In addition, NCD risk factors exclusively contributed to a lower HRQOL between PLWH, after controlling for socio-demographic variables and HIV-related factors (disclosure of HIV status, CD4 cell count, and the length of HIV). This means that prevention and control of NCDs and their risk factors is an important public health concern in HIV care and treatment. While tobacco use and abdominal obesity were associated with a poor mental HRQOL, physical inactivity and hypertension were associated with a poor physical HRQOL. Findings highlight the importance of lifestyle modification, including regular physical activity, healthy eating habits, and smoking cessation programmes in PLWH. Adopting a healthy lifestyle is viewed as one of the best ways to address risk factors for NCDs, which in turn improves the HRQOL of PLWH.

These results also confirm previously reported results. A negative association was found between tobacco use and the HRQOL, especially the mental HRQOL (Kowal et al., 2008; Turner et al., 2001). The relationship between tobacco use and a bad mental HRQOL is evident due to the growing body of evidence that shows that smokers are more likely to have psychiatric co-morbidity (Shuter, Bernstein, & Moadel, 2012). Tobacco use is also well-acknowledged as one of the most important modifiable risk factors for different conditions, including pulmonary tuberculosis, CVDs, and AIDS-related cancers (Shirley, Kaner, & Glesby, 2013) and more as a predictor of low life expectancy than HIV itself in PLWHI (Helleberg et al., 2015). Thus, a decline in the HRQOL and increases in smoking-related mortality can be expected in PLWH of all ages. This implies that health promotion programmes that focus on smoking prevention and cessation should be prioritised in this population. Interestingly, in this study no association was found between tobacco use and the physical HRQOL. This is despite the availability of evidence on the detrimental effects of tobacco use on the immune system and ART (Miguez-Burbano et al., 2003; Shirley et al., 2013). Despite this, other researchers suggest that a lower HRQOL in PLWH might be attributable to chronic obstructive pulmonary disease rather than tobacco use itself (Drummond et al., 2010). In light of this research, well-designed research is needed to clarify the relationship between tobacco use and a HRQOL.

Additionally, physical inactivity was associated with a poor physical HRQOL. These findings are consistent with previous studies (Kowal et al., 2008; Uphold, Holmes, Reid, Findley, & Parada, 2007). There is also much evidence to show positive effects of physical activity in PLWH, including physical and psychological benefits (Derman et al., 2010). In contrast to the current study, Uphold et al. (2007) found a relationship between healthy diet and a physical and mental HRQOL. Thus, interventions to increase physical activity in PLWH are warranted. Physiotherapists are in a better position to design programmes to increase PLWH participation in physical activity and to increase awareness of the benefits of physical activity among other health care services.

Furthermore, abdominal obesity and hypertension were associated with a reduced physical and mental HRQOL. A growing body of evidence indicates that abdominal obesity is prevalent among HIV+ adults on antiretroviral therapy (Hejazi, Huang, Lin, & Choong, 2014). These suggest that targeting abdominal obesity in the care of PLWH could provide a better overall QOL, especially for those living with HIV. Existing evidence has also shown that hypertension is strongly associated with cardiovascular and kidney disease among PLWHI (Peck et al., 2014), which in turn may be associated with a lower QOL in PLWH. Thus, screening and treatment of hypertension must be an integral part in following-up with PLWH.

Findings also reveal the significance of socio-demographic and HIV-related factors to predict the HRQOL of PLWH. Lack of disclosure of HIV serostatus to somebody was associated with a poor mental HRQOL. The same results were found in previous research (Bunjoungmanee, Chunloy, Tangsathapornpong, Khawcharoenporn, & Apisarnthanarak, 2014). These findings provide an opportunity to improve the HRQOL for those living with HIV. In HIV, disclosure specifically relates to informing others of one's positive HIV status − primarily to one's sexual partner(s), but broader definitions encompass disclosure to family members and other social networks. Health care professionals are required to assist PLWH in the process of making the decision as to whether or not to disclose their HIV serostatus.

The available evidence also indicates that there is an association between HIV status disclosure and social support. Social support may facilitate disclosure among family members (Go et al., 2016; Murphy, Moscicki, Vermund, Muenz, & Adolescent Medicine HIV/AIDS Research Network., 2000) and in turn improve self-esteem, coping, and engaging in healthy lifestyle behaviours (Atuyambe et al., 2014). However, there is no common formula for disclosure counselling; it varies from context to context, and, importantly, from person to person. Sometimes disclosure is a person's choice, whereas in other circumstances, PLWH are forced or obliged to disclose their seropositive status due to varied HIV-related symptoms that they may be experiencing. In this regard, effective disclosure counselling should consider a person's psychosocial context, including their social support networks, the stigma, depression, or occupation-related health issues. In this regard, the training of health care providers on how to assist PLWH with disclosure decisions and resources are central to good care. These are viewed as effective strategies to help out PLWH. Further research is needed to develop evidence-based, disclosure-focused screening questions to identify those who need special attention in HIV disclosure counselling.

Being unmarried was associated with a poor mental and physical HRQOL. These results are in accordance with previous studies that indicate that a stable relationship contributes to a physical HRQOL (Perez et al., 2005; Préau et al., 2007). These results re-affirm the importance of social support in enhancing the HRQOL of PLWH. Apart from family members and friends of PLWH, this research's findings support HIV support groups being integrated into social networks for PLWH to assist, especially those who perceive themselves to have a low level of social support. Once again, participants with a lower CD4 count also reported a poor physical HRQOL. These findings are consistent with other studies on the topic (Kowal et al., 2008; Liu et al., 2006). Armon and Lichtenstein (2012) found further evidence of association between CD4 cell count and mental HRQOL. This study's results suggest that the initiation of ART to all PLWH, regardless of their CD4 cell count, can reduce morbidity and mortality, and improve the QOL of PLWH. Thus, intervention strategies to improve ART adherence in PLWH are warranted.

These results suggest that the assessment of associations between the behavioural and biological risk factors for NCDs and an HRQOL provides opportunities for targeted counselling and secondary prevention efforts, so that health care providers implement strategies that have a significant impact on the HRQOL. Modifiable and preventable factors associated with the HRQOL provide potential targets for intervention. These findings highlight the importance of a multidisciplinary care approach in the care of PLWH, including dieticians and physiotherapists, to implement effective, healthy lifestyle interventions. The association of modifiable and preventable risk factors provides an opportunity to enhance the physical and mental HRQOL in PLWH. In this regard, efforts to support inter-professional, collaborative practice and person-centred, integrated service delivery are the key to interventions, so as to promote lifestyle changes for NCD risk factor reduction in PLWH. It is apparent that a comprehensive health care of PLWH should offer a continuum of care with a multidisciplinary team to deal with the needs and various characteristics of PLWH. The role of each team member should be defined in a more proactive way. Physiotherapists, as exercise experts, need to establish appropriate physical activity guidelines for PLWH, and physical activity would be part of a routine health assessment in HIV clinics. In this regard, national programmes should be adopting programes to improve physical activity and comprehensive multidisciplinary programmes in response to evidence from studies done previously and this important study too.

Even though it is very hard to change one's behaviour, the current study indicates the importance of promoting counselling on the adverse outcomes of NCD risk factors and the benefits of engaging in preventive health behaviours. Apart from that, a health promotion approach will help to empower people and involve them in decisions about health behaviours to reduce their NCD risk. Addressing a single risk factor without considering multiple risk factors for NCDs and other modifiable NCD risk factors is an inefficient or unsustainable approach, hence, consideration of both behavioural and biological risk factors may help to reduce the burden of NCD risk factors, and result in optimal outcomes for PLWH.

### Limitations of the study

There were a number of limitations to this study. This current study only focused on PLWH adults (individuals over 18 years old), the participants were not randomly selected from the population, and the recruitment of the participants occurred in health centres only; therefore, the sample may not be generalisable to the total the population of PLWH in Rwanda.

## Conclusion

The results reveal the moderate physical and mental health-related dimensions of QOL among PLWH in Rwanda. While the results indicate that tobacco users and those who had abdominal obesity reported a poor mental HRQOL, physical inactivity and hypertension have a negative impact on the physical HRQOL. In addition, certain socio-demographic and HIV-related variables – specifically being unmarried, lack of HIV disclosure, and low CD4 count (less 350 cell count/mm^3^) – were associated with significantly lower mental and physical dimensions of QOL. The association of behavioural and biological risk factors for NCDs with a poor physical and mental HRQOL suggests that there is still impairment in the HRQOL of those living with HIV with access to ARV drugs in Rwanda. This provides a window of opportunity to improve HRQOL; thus, intervention strategies to prevent and control NCD risk factors should be viewed as central to PLWH. Given that behavioural and biological risk factors are preventable and modifiable, risk factor modification should be ensured with appropriate education programmes for PLWH, with the emphasis on reducing and controlling NCD risk factors in order to improve the HRQOL of those living with HIV, and should be accompanied by regular monitoring of these risk factors. Health care providers should be provided with the necessary information to help PLWH who are more vulnerable to NCDs due to the HIV itself, drugs’ side effects, and an unhealthy lifestyle. Assessing the epidemiology of preventable risk factors for NCDs in PLWH is the basis of prevention of NCDs in this population. It may help HIV healthcare providers and policy-makers to design and target intervention programmes for preventing and controlling NCDs.

## Availability of Data and Material

The datasets analysed during the current study are available from the corresponding author on reasonable request.
